# Identifying Emerging Motif in Growing Networks

**DOI:** 10.1371/journal.pone.0099634

**Published:** 2014-06-17

**Authors:** Haijia Shi, Lei Shi

**Affiliations:** State Key Joint-Laboratory of Environmental Simulation and Pollution Control, School of Environment, Tsinghua University, Beijing, China; Université de Nantes, France

## Abstract

As function units, network motifs have been detected to reveal evolutionary mechanisms of complex systems, such as biological networks, food webs, engineering networks and social networks. However, emergence of motifs in growing networks may be problematic due to large fluctuation of subgraph frequency in the initial stage. This paper contributes to present a method which can identify the emergence of motif in growing networks. Based on the Erdös-Rényi(E-R) random null model, the variation rate of expected frequency of subgraph at adjacent time points was used to define the suitable detection range for motif identification. Upper and lower boundaries of the range were obtained in analytical form according to a chosen risk level. Then, the statistical metric Z-score was extended to a new one,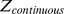, which effectively reveals the statistical significance of subgraph in a continuous period of time. In this paper, a novel research framework of motif identification was proposed, defining critical boundaries for the evolutionary process of networks and a significance metric of time scale. Finally, an industrial ecosystem at Kalundborg was adopted as a case study to illustrate the effectiveness and convenience of the proposed methodology.

## Introduction

Network motifs have been widely identified as basic building blocks of many complex networks, such as biological networks [1]–[3], food webs [1], [4], [5], engineering networks [1], [6], and social networks [7]–[9]. Compared with the node level and component level, motifs can present more information about how basic elements of networks interact with each other to let different system functions emerge [10]–[15]. For example, in studies of the hierarchy structure of a protein-protein interaction network, more importance is attached to network motifs than nodes [16]. And motifs are found as a good way to simplify the description of network structure [17].

Being building blocks, motifs are widely thought to contribute to the stability of existing networks by carrying out specific functions. Prill et. al proved that the robustness of biological networks to small perturbations is highly correlated with the relative abundance of network motifs. They thought that the robust dynamical stability plays a key role in the evolutionary process of the non-random structure of biological networks [3]. Similar results were also obtained in regulatory networks [18], [19]. More specifically, ordered cyclic motifs, not only in biological networks, but also in engineering networks, were found providing dynamic stability [20]. In ecology, Stouffer et al. empirically demonstrated that the prey selection mechanism among species is consistent with the properties of the over- and under-representation of the ‘food-web’ motifs [5]. And, the network motifs, predator-prey loops, cascade into the stability of the whole food web [4], [21].

Motifs have also been considered to be structural carriers of evolution mechanisms of networks. In studies of biological evolution, conservation usually implies importance. The conservation of the proteins in a motif is conjectured to be indicative of the biological importance of that motif [16], [22], [23]. Similar results also appear in the gene regulation networks of Escherichia Coli and Saccharomyces Cerevisiae [24]. The relation of emergence of motifs and mechanisms of networks has been attracting increasing attention, especially from the perspective of network evolution. Scholars argue about which mechanism has contributed to these overrepresented sub-structures: structural preference, duplication of ancestor circuits, optimal design, or natural selection and try to explain the origin of modularity and network motifs in biology [16], [22], [24]–[30]. Emergence of motifs in the evolutionary process can be regarded as a key indicator on the meso scope. In other words, it means that the footprints of evolutionary events of many systems are suggested to be traced by network motifs [31]–[34]. Moreover, network motifs in the evolving systems in other disciplines also cause wide concerns. Kaluza et al. find that robust motifs emerge from the evolutionary process (against structural noise signal) of flow distribution networks [35]. Hales and Arteconi show that the four-node undirected motif distribution of the network of cooperation between selfish nodes in a network produced by peer-to-peer protocols kept stable at three discriminate stages of the evolutionary process [36]. Squartini and Garlaschelli report the motif distributions of the world trade network from 1950–2000 and find that the dyadic structure of this system carry main information of evolutionary process rather than triadic motifs, the significant profiles of which have almost kept stable in this process [9]. By fully taking into account the longitudinal dimension, Bajardi et al. take dynamical motifs to uncover the network evolution of cattle trade movements and contribute to control measures for zoonotic diseases [34].

Roughly speaking, network motifs can reveal evolutionary mechanisms of systems. However, emergence of motifs in growing networks may be problematic. According to the definition of motif, it is a kind of small connected substructure made of 2–20 nodes whose occurrences in the observed networks are significantly higher than the expectation in their random counterparts [1]. In the initial growth stage of a given network, the frequency of every subgraph will perhaps fluctuate violently with the addition of edges one by one. The statistical metrics of some graphs may have been relative significant for a long time, but others may be significant only at some single time point. It is essential to discriminate them. For a given network, will an observed motif always be statistically significant in the initial growth stage? In other words, it is necessary to confirm the critical time of the evolutionary process when the identification results of network motifs start to be trustworthy.

Furthermore, there exist some small scale networks in some disciplines, such as food webs, social networks and industrial networks that are constrained by space. Unlike networks with thousands to millions of nodes, these networks usually consist of dozens of nodes and edges. Many motif detection methods assume that the degree distribution of networks would follow some standard ideal distribution types, like random, power-law, or exponential distribution, when estimating the concentration of subgraphs [37]–[42]. But this hypothesis is usually invalid before the network grows to a certain size. It is quite hard to control the error bounds [43].

In all, for growing networks or small scale networks, two questions should be answered: 1) what is the critical size of target network that can make the results of motif identification stable and meaningful? 2) how to identify the stable motif from a group of candidate subgraphs in the evolutionary process of networks? This paper contributed to the two questions, and is organized as follows: followed by the introduction part in Section 1, Section 2 provides a modified analytical framework of motif detection, including a method to determine the critical network size and a new statistical metric to evaluate the persistence of the appearance of network motifs. A case study is introduced in Section 3, which covers the evolutionary process of an industrial ecosystem over 50 years. Section 4 uses this case to illustrate the proposed detection procedure. By testing the network robustness under different degree’s random disturbance, the validity of the motif identification methodology in this case is discussed in Section 5.

## Methods

The traditional framework of detecting network motifs is usually divided into four parts applying different procedures:

1. To count the frequency of each subgraph or a given one in the investigated network,
2. To generate randomized networks by an appropriate null model,
3. To decide whether subgraphs are topologically equivalent or not and classify the isomorphic ones into the same group,
4. To determinate the statistical significance of each subgraph.

On the premise of acceptable detection accuracy, much time and effort was spent on the generation of randomized counterparts and eliminating biased sampling of this ensemble, modifying the reasonability of the null model to exclude the influence of types of constraints, improving the operation efficiency and scalability of detection algorithms to match the need of finding motifs of larger size and saving storage memory usage. The details of every aspect of motif detection were reviewed comprehensively in these references [1], [39], [42], [44]–[51].

For the first question, it seemed to be quite complicated to design a special procedure for each small scale network, because the network size of each of them was usually small so that the degree distribution was not smooth enough to fit standard degree distribution types well. Thus, to simplify the procedure of solution-finding and to make it universal, the null model used to generate randomized network was proposed to fix the degree sequence of investigated topologies.

Methods of motif detection are mainly based on two different strategies: (1) to compare the concentration of subgraphs with the corresponding expected values of the ensemble of randomized networks generated by an appropriate null model, (2) to compare the concentration of subgraphs with the corresponding expected values in a well-chosen probabilistic model of degree distribution, such as the power-law or Poisson distribution [2], [42], [52].

The methodology for motif detection was based on statistical theory. The larger the network size was, the more reliable the detection result was. If a new connection was added to a network, the new result of motif detection might deviate significantly from the original one. Therefore, it was necessary to give a reasonable critical value of network size so that the identified motifs made sense. Then, under the guide of the estimation formulas for the concentration of subgraphs in E-R random network model [53], a persuasive procedure was designed to give a reasonable answer to the first question, shown as below.

Let us consider the E-R random network model with the number of node *N*, and the number of edge *E*. There are all three different placements for the three possible types of edges between any two vertices *i* and *j*: a unidirectional edge *u*, a bidirectional edge *b* or nonedge *n*. The connection density of the network *p* is defined as
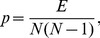
(1)


The probabilities for each of the three connection status is given by
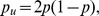


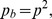


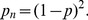
(2)


**Figure 1:**
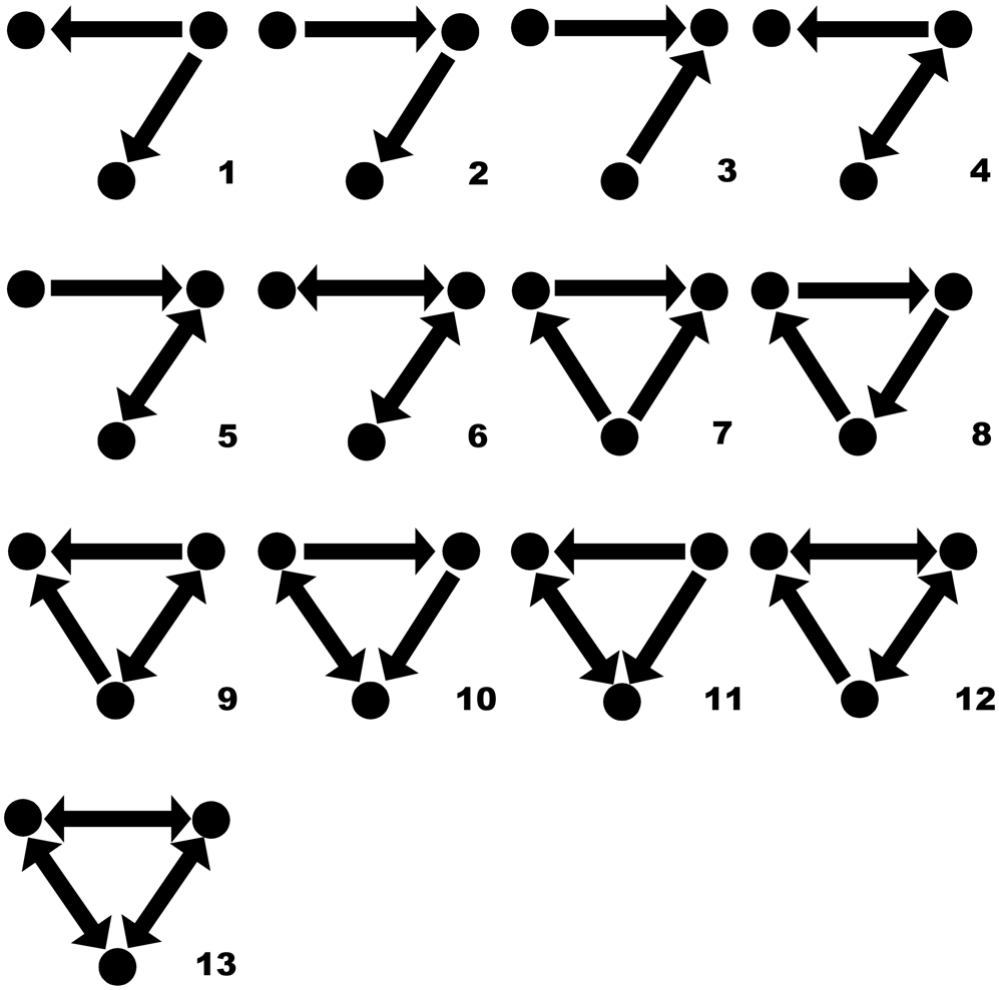
All connected directed subgraphs of size three.



According to the amount of these three connection status in subgraphs of size three, all thirteen types of subgraphs, shown in Figure 1, are divided into seven template types. The expected value 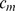 of subgraph type *m* is given in reference [53] as below:

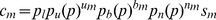
(3)Where the number of possible placements for a subgraph of size three is 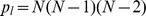 and 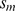 is the symmetry factor. Values of 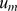, 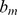, 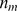, 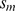 for each subgraph type are listed in Table 1. The detail for the derivation process of 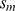 was described in reference [53].

**Table 1:** Number of unidirectional edges *u_m_*, bidirectional *b_m_*, and nonedges *n_m_* and the symmetry factors *s_m_* of all three-vertices subgraphs.

template	1			2		3	4		5			6	7
subgraph	1	2	3	4	5	6	7	8	9	10	11	12	13
*u_m_*	2	2	2	1	1	0	3	3	2	2	2	1	0
*b_m_*	0	0	0	1	1	2	0	0	1	1	1	2	3
*n_m_*	1	1	1	1	1	1	0	0	0	0	0	0	0
*s_m_*	1/8	1/8	1/4	1/2	1/2	1/2	1/8	1/24	1/8	1/8	1/4	1/2	1/6

Note: the mistakes of the value of *s_m_* in the expression for symmetry factors and also the values in reference [53] had been modified here.

Let us consider a simple case in the E-R random null model: give the amount of nodes *N* and that of directed edges *E*. For any subgraph *g*, its expected number of appearance 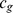 in the ensemble of randomized networks 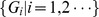 generated by this null model can be calculated by the formula (3). Now, when a new edge is added into 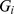 at a time point *t*, the network changes from state *G*(*t*) to another state *G*(*t+*1). At the same time, the expected number 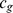 also changes from 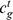 to 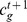. Let its change ratio be 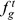, so
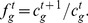
(4)


In this paper, the starting time for the procedure of motif detection in a growing network is proposed to be given from the time scale, which is shown as follows: If the variable 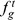 of subgraph *g* meets the condition,
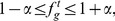
(5)it will be checked as a motif or not. The value of 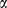 depends on the corresponding acceptable level of risk, for instance, 0.01, 0.05 or 0.1. It is necessary to emphasize that each of the seven subgraph templates should be checked separately, because their function types of 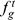 are different from each other.

**Figure 2:**
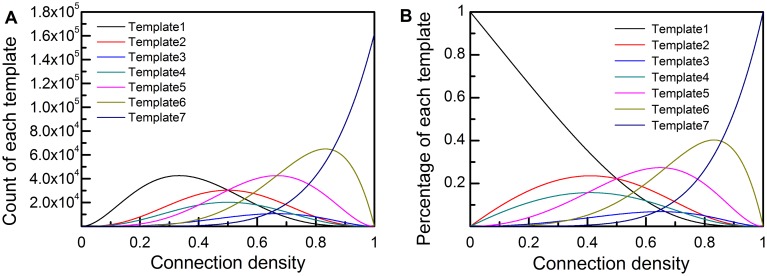
The amount and the percentage of each subgraph template in a randomized network with *N* = 100. (**A**) The change of the count of each subgraph template, with the connection density 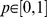. (**B**) The change of the percentage of each subgraph template with 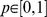.

For a given set of vertices, with the growth of connectivity *p* (from 0 to 1), and also the edge amount (from 0 to *N*(*N*-1)), the change of 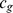 and 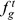 of all seven subgraph templates are shown in Figure 2. Both the frequency amount and the percentage of each template are given and compared in a growing network with *N* = 100.

It is found that, within the range of 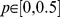 in the E-R random null model, which covers the connectivity of most study cases about network motifs, the abundance of template 1 is much higher than others. It reaches the peak at *p* = 1/3. In the whole range, its percentage keeps decreasing monotonically from 100% to zero. All the first six subgraph templates will be transformed to the seventh one, the fully-connected subgraph. Thus, it may be conjectured that for most real networks, subgraphs belonging to template 1 should be probably of the highest concentration.

Because of the similarity of function types of the frequency of these seven subgraph templates, just some differences in parameters, the variation of 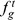 at any two adjacent time points can be simplified to five different situations: (1) Template 1, (2) Template 2 and 4, (3) Template 3 and 5, (4) Template 6, (5) Template 7. Then, the analytical result for template 1 is taken as an example to illustrate the procedure of defining the suitable detection range of motif identification in terms of time.

**Figure 3:**
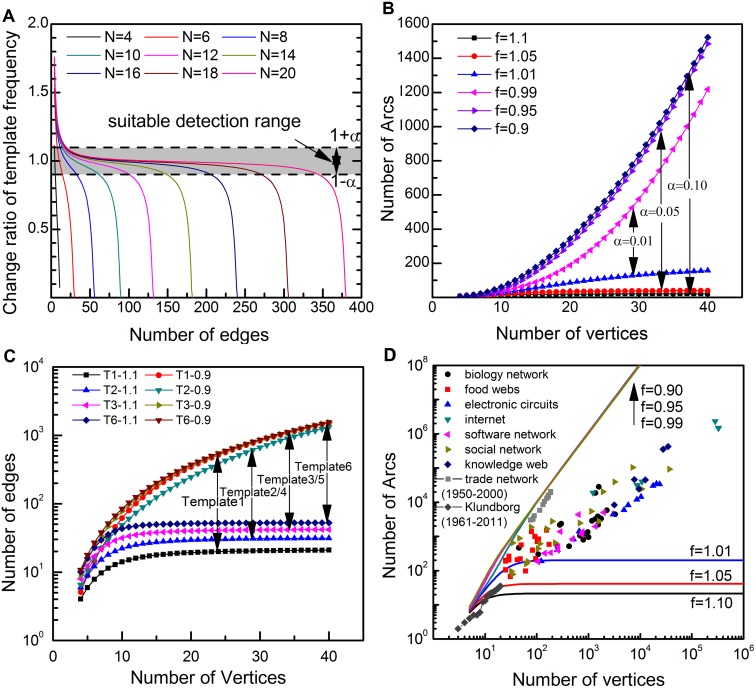
Defining suitable detection range for different subgraph templates based on E-R random null model. (**A**) The rate of frequency change of subgraph template 1 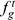 in growing networks with fixed number of vertices *N*, which varies from 4 to 20. The range between 1±α is fielded in gray. α = 0.1. (**B**) The detection range of subgraph template 1 for networks of different size and connectivity. (**C**) The detection range for different subgraph templates (T1–T6). α = 0.1. (**D**) Networks from multidiscipline are compared with the suitable detection range for subgraph template 1.

The variation of 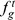 of template 1 in networks of different size which are generated by random null model is shown in Figure 3(A). Each curve records a growing process of a network composed by a set of vertices in the range of 4–20. Let 1±α (α = 0.10) be the acceptable variable range of 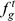 for motif identification, which is fielded in gray. Then the upper and lower boundaries of this range are given in the forms of analytical solutions. It is evident that the larger *N* of a network is, the wider this range is. Meanwhile, when α is equal to 0.05 or 0.01, the corresponding functions of upper and lower boundaries are also given, shown in Figure 3(B). It is indicated that with the growth of α, this range becomes wider and wider.

In our opinion, if the size of a network, represented by *N* and *E*, locates in the area between the upper and the lower boundary, this network should be thought reasonable to check whether the subgraph belonging to the template type is a motif or not. According to the equation (1), (2), (3), (4), (5), the analytical solutions to the upper and lower boundary of each template are calculated, given in Table 2. All seven templates have the lower boundary, which is expressed by a quadratic equation about *N* and *E*, and all of them except template 7 have the upper boundary, which is expressed by a kind of logistic function. Both the two function types are shown in Table 2, also with the corresponding parameters for each subgraph template. The risk level α is equal to 0.10, 0.05 or 0.01 separately. The detection ranges for the first six templates are compared in Figure 3(C). These ranges partially overlap and they have a common area. And it is obvious that the range of template 1 covers the widest interval. To be simple, this paper proposes that the widest range or the narrowest one can be regarded as the detection range for motifs detection in all networks. It can also be treated as the necessary conditions for the existence of network motifs.

**Table 2:** Analytical solutions to the suitable detection range of all subgraphs templates.

Category	1–α	y = A+Bx+Cx^2^			1+α	y = A+B/(1+(x/x_0_)^p^)			
		A	B	C		A	B	X_0_	P
**Template1**	**0.90**	–5.716	–3.307	1.040	**1.10**	21.34	–21.27	7.571	2.324
	**0.95**	8.301	–6.694	1.094	**1.05**	41.07	–40.66	10.52	2.338
	**0.99**	84.78	–17.73	1.144	**1.01**	201.1	–200.1	22.93	2.266
**Template2/4**	**0.90**	–3.092	–2.735	1.030	**1.10**	31.69	–31.54	7.257	2.533
	**0.95**	6.523	–5.051	1.065	**1.05**	61.18	–60.15	10.07	2.557
	**0.99**	55.15	–12.26	1.091	**1.01**	303.0	301.3	21.95	2.429
**Template3/5**	**0.90**	–1.401	–2.150	1.019	**1.10**	42.03	–41.09	6.971	2.882
	**0.95**	4.509	–3.576	1.040	**1.05**	81.31	–78.65	9.620	2.904
	**0.99**	32.99	–7.908	1.053	**1.01**	407.0	–400.4	20.88	2.675
**Template6**	**0.90**	–0.2900	–1.568	1.009	**1.10**	52.46	–48.76	6.742	3.641
	**0.95**	2.456	–2.233	1.019	**1.05**	101.7	–95.32	9.163	3.588
	**0.99**	15.25	–4.224	1.024	**1.01**	514.6	–499.9	19.69	3.129
**Template7**	**0.90**	–	–	–	**1.10**	63.45	–	–	–
	**0.95**	–	–	–	**1.05**	123.5	–	–	–
	**0.99**	–	–	–	**1.01**	603.5	–	–	–

Note: y represents the number of edges in the network, and x represents the number of vertices.

In spite of great difference in the size of natural or artificial networks, the birth and growth of every network should start from scratch. It means that there should be a starting point in the growing process of networks, when motifs may emerge from common subgraphs and the significance can maintain stability for a while. Unfortunately, for various reasons, the time series data of the whole evolutionary process of most networks are too difficult to collect.

Motifs have been identified in plenty of networks in research fields of biology, ecology, engineering, social science and many other artificial systems [1], [5], [6], [9], [54]–[60] in the past few decades. The size of previously studied networks are marked in Figure 3(D), in contrast with the upper and lower boundaries (α = 0.01, 0.05, 0.10) proposed in this paper. The size of social networks and food webs are relatively small, containing just dozens of vertices and edges. The evolutionary process of Kalundborg industrial ecosystem, our case study, is also shown with black diamond.

Another important question for motif identification in the evolutionary process of networks is how to identify stable motifs that have continuous statistical significance from candidate subgraphs. In other words, if the significance of some subgraph is intermittent in time, it will not be considered as a stable motif in terms of time. Because each metric used to measure the statistical significance of subgraphs is based on statistical theory, the values of motifs should be of high correlation at adjacent time points of the evolutionary process, without serious fluctuation. The more significant the metric is, the stronger this correlation is, and the more reliable the result of motif identification is. In general, the metric designed for time series analysis needs to be measured not only for the statistical significance at a single time point, but also for that of a certain period of the whole evolutionary process.

Now let 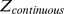, based on *Z*-score at each single time point of a continuous period, be the statistical metric to measure the significance of subgraphs:
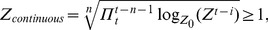
(6)where 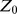 is the significant threshold of the metric Z-score. *t-n*, *t-i*, and *t* are different time points of the evolutionary process of the investigated network. So 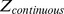 is the metric which can reflect the average level of the significance of a subgraph in a continuous period of time.

A random experiment is designed to demonstrate the validity and practicality of this new metric. In the same experiment, the optimum value of the parameter *n* can be inferred. Though the experiment is based on E-R networks, the time series data generated by this experiment has certain representativeness about the fluctuation of Z-score values in real networks. Its positive significance lies in offering a kind of thought and an operation method to optimize the parameter *n* in eq. 6.

**Figure 4:**
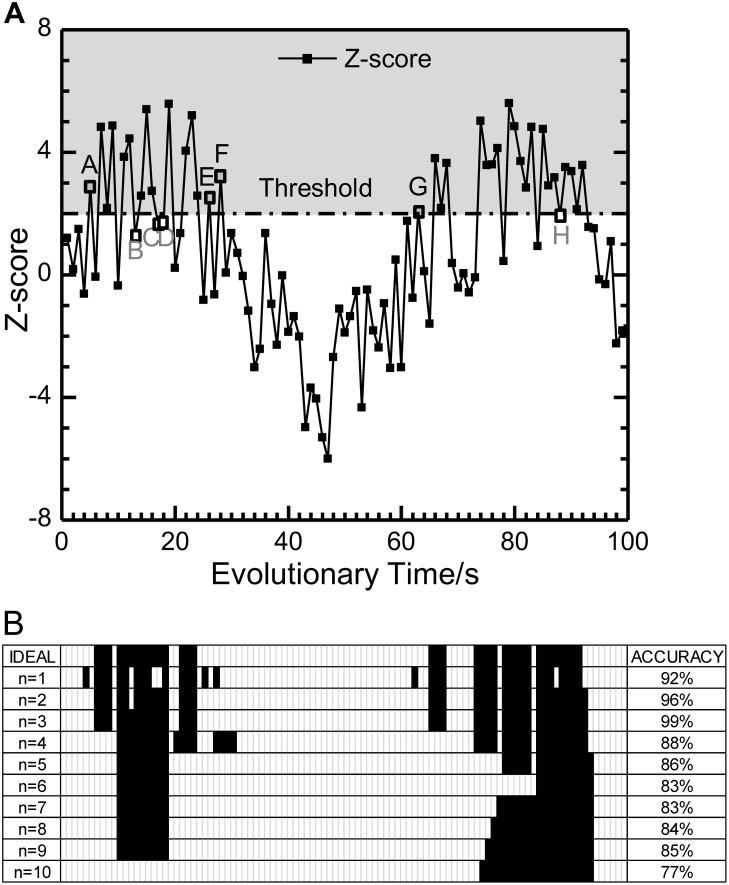
Results of motifs identification in a random experiment. (**A**) The variation of the significance metric Z-score in the whole evolutionary process. (**B**) Identification results of motifs when *n* is equal to 1, 2……10, separately. The accuracy of each *n* is compared with the ideal result in the first row.



In order to simulate the temporal variation of the metric Z-score of subgraphs in real networks, 100 random numbers around the statistical threshold (let 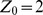) are generated by the function (7) as below, which is designed to be of both the time continuity and randomness, shown in Figure 4(A):

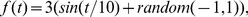
(7)where *t = *1, 2…, 100 represent time points of evolutionary process of networks. And random(–1,1) means random number between −1 and 1.

The gray part represents the significant area of Z-score. According to the principles for motif identification process in time series data proposed above, the statistical significance should be continuous in time. In other words, in the growing process of networks, the statistical significance of each time point should be reappraised by Z-score values of its neighbor time points. If there is an isolated significant or non-significant time point in a period, its feature will be replaced by the average level of its neighbors’. According to this criterion, the ideal identification result (Ideal) of significant range can be given. In Figure 4 (A), although points A, E, F and G are located upon the threshold line, points B, C, D and H are located below it, the corresponding identification results should be reversed, referring Z-score of the adjacent time points around each of them. Set this ideal identification result as the benchmark, then compare this ideal result with that calculated by formula (6), where *n = k*, *k* = 1, 2…, 10. The time points of the appearance of motifs in the evolutionary process are marked in black, shown in Figure 4(B). The accuracy of the identification result by 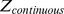 for each *n* is given in the last column. It is found that the identification result of *n* = 3 is of the highest accuracy, and that of *n* = 2 is second highest, while that of *n* = 1 is just 92%. Therefore, the recommended value of the parameter of *n* in formula (6) is 3. It is concluded that 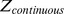 performs better than Z-score based on single point when we analyze time series data of growing networks.

## Materials

Our case study investigated an industrial ecosystem at Kalundborg in Denmark, which has evolved for more than fifty years since 1961. Many cooperative relationships among enterprises were established by reusing or recycling of industrial wastes and sharing infrastructure services.

**Figure 5:**
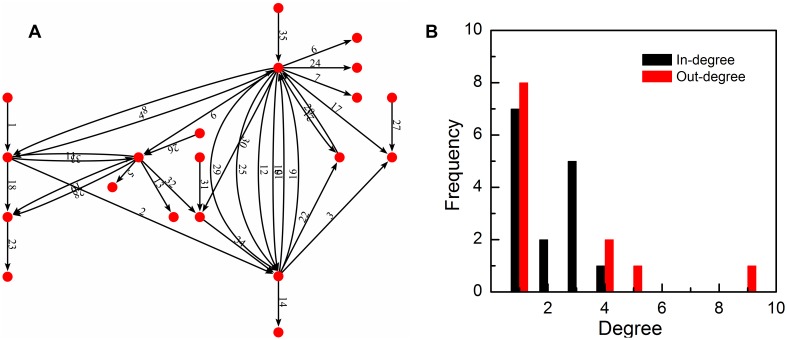
The structure and degree distribution of the industrial network at Kalundborg. (**A**) Red vertices represent enterprises and links represent material and energy flow. The chronological order of these directed edges are marked with the serial number 1, 2…, 35. (**B**) The in-degree and out-degree distribution in 2011.

In this industrial network, enterprises were abstracted into vertices and material and energy flows between each pair of them were abstracted into directed edges. By now, there have been 20 vertices and 35 directed edges. Its growth process is shown in Figure 5 (A), in which the chronological order of these directed edges are marked with the serial number 1, 2…, 35. Multiple edges were conserved in the description of networks, but simplified in the process of motif detection. All the information about our case was obtained from the official website of Kalundborg symbiosis: http://www.symbiosis.dk/en/system.

Before applying the framework based on E-R random model to identify network motifs in our case study, we examined its in-degree and out-degree distribution in 2011 (shown in Figure 5 (B)) to verify that the application network is of E-R type, Scale-free type or neither. Chi-square test was used to examine the hypothesis that the in-degree or out-degree follows a Poisson distribution, while K-S test [61] was used to quantify if the in-degree and out-degree of application network are drawn from power-law distribution. The in-degree data passed the chi-square test, but the out-degrees did not (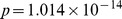). For K-S test, both the in-degree and out-degree data passed, with power exponent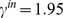, 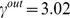, and 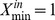, 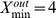, respectively. It was noticed that although in some situations the degree data passed statistical tests, there are only four data points of degree values in the dataset of in-degree distribution and also out-degrees’. For networks of small scale, the dataset was not big enough to fit some classical degree distribution well. In our research, E-R random model was regarded as a default model to describe small scale networks.

An ensemble of 1000 randomized networks with the degree sequence given by the investigated network was generated to calculate the expected value and the standard deviation of the frequency of each subgraph of size three. “Switching” strategy was used to realize the randomized process: each edge was exchanged 10 times and each exchange attempted 10 times. FANMOD was used to identify motifs in the case study based on the parameters introduced above [48]. During the randomized process, unidirectional edges were only exchanged with unidirectional ones. The same applied for bidirectional edges. Therefore, the number of incident bidirectional edges remained constant for each vertex. Both the metric Z-score and 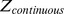 (*n* = 3) were used to measure the statistical significance of all subgraphs. As assumed and proven by many studies except for some networks in biology, the distribution of the frequency of subgraphs in the ensemble of randomized networks generated by null models fit the normal distribution well, the thresholds of “Z-score” are set at “1.281”, “1.645”, and “2.326” under the different confidence level “90%”, “95%” and “99%”[50]. For our case study, the threshold of Z-score was set to 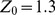. The threshold of the frequency of motifs was suggested to be 5.

## Results

**Figure 6:**
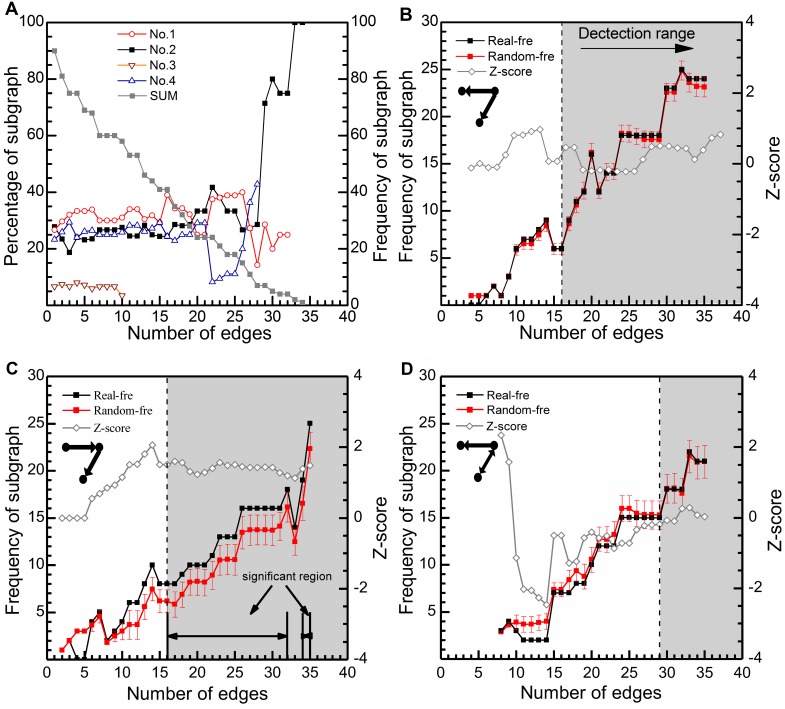
Identifying motifs in the evolutionary process of the industrial ecosystem at Kalundborg from 1961–2011. (**A**) The variation of the percentage of four subgraphs (No.1–4) and that of all three-vertices subgraphs. (**B**) The result of subgraph No.1. The suitable detection range is the right area in gray. Its frequency is compared with that of the average level in the ensemble of 1000 randomized networks. The error bar, marked by red sticks, represents the standard deviation. The variation of statistical metric Z-score is marked by hollow black box. (**C**) The result of subgraph No.2 and the corresponding significant region. (**D**) The situation of subgraph No.4.

Within this growth process of the industrial network at Kalundborg, the sum of the frequency of subgraphs has also been increasing. By 2011, ten types of subgraphs (No.1–7, 9, 10 and 12 in Figure1.) have been found in the evolutionary process, but only four of them (subgraph No.1–4) have appeared more than four times (the frequency threshold of network motifs). The variation of the percentage of these four subgraphs and the sum of the frequency of all three-vertices subgraphs, marked in gray, are shown in Figure 6(A). It is obvious that the percentage of subgraphs (No.1, 2, 4) tends to be stable after the appearance of the fifteenth edge, within the range of 20%∼40%. But before that, these curves fluctuate quite sharply.

According to the method of motif identification proposed above, it was necessary to first define the suitable detection range for each subgraph in this case. Subgraph No.1 and 2 belonged to template 1, while subgraph No.4 belonged to template 2. Let α = 0.1. Then, reading from Table 2, the corresponding upper and lower boundaries for each template are separately listed in Table 3:

**Table 3:** Detection boundaries for subgraphs in the industrial ecosystem at Kalundborg (α = 0.1).

Boundary	Template1	Template2
	subgraph No.1, 2	subgraph No.4
**Upper**	y = −5.716–3.307x+1.040x^2^	y = 21.34–21.27/(1+(x/7.571)^2.324^)
**Lower**	y = −3.092–2.735x+1.030x^2^	y = 31.69–31.54/(1+(x/7.257)^2.533^)

Plug the growth data of the industrial network into functions (4,5) and then the critical size of it for each template is obtained. The suitable detection range of template 1 starts from N = 11 and E = 16. By contrast, the range of template 2 is much narrower, starting from N = 17 and E = 29. With the formula (6), the significant range of subgraphs No.1, 2 and 4 are calculated and shown in Figure 6(B), (C) and (D), with the variation of the corresponding Z-score. The frequency of each subgraph and that of the average value of 1000 randomized networks are compared. The error bar represents one time the size of standard deviation, marked in red. Among the three subgraphs, only No.2 is the significant motif which covers all suitable detection range besides N = 19, E = 33. By analyzing the variation of Z-score, it is seen that in the first half growth stage of the network, this statistical metric fluctuates irregularly, while in the second half stage, its value tends to be stable, by comparison. This common phenomenon also means that with the growth of this industrial network, the identification results turn to be more and more trustworthy and it is necessary to set the detection range for motif identification, just as was proposed in the method section.

In order to illustrate the reliability of the result of motif identification in our case, another random experiment is designed. A new metric named the combination of subgraphs’ frequency (CSF) is proposed here: for a given network *G*, the frequency of every subgraph of size *k* can be enumerated as: 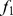, 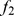
…, 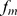. Then, these data make up a sequence of frequency, which is named the sequence *Q*. It is unique for a given network. But for the same 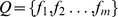, it may correspond to different network topologies.

For the whole evolutionary process of a growing network, assume that the number of vertices *N* and that of edges *E* have always kept increasing. When 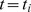, the three measurements of the network 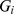 are 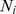, 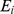 and 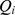, respectively. We can learn what happened to the growth process of 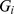 by investigating the differences of 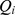 in an ensemble of randomized networks that are generated by operating the switching algorithm for several times on 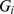. In other words, it can tell us the necessity of defining the suitable detection range for motif identification.

The detail of this randomized experiment is clarified as following:

1. For a given network 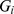 with 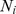 vertices and 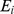 directed edges, randomly select two directed edges a→b and c→d in it, then exchange their ends to form two new edges a→d and c→b, repeat this procedure for *n* times to generate 1000 randomized networks: 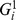, 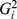
…, 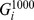. The switching times *n* ranges from 1 to 10*E*.
2. Enumerate all different 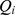 in the ensemble of 1000 randomized networks {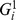, 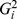
…, 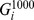}. Record the sum value as the metric 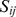 when *N* = *i* and *E* = *j*.
3. Fix 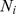, and increase 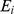, then, record the variation of 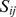 at every time point.
4. Let *i* = 1, 2…, 35. Repeat (1–3) at different *i*.

In our research, the metric 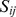 is regarded as another type of measurement of the randomized degree of the given network 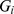, corresponding to the switching times *n*. This view can be explained as follows. With the growth of *n*, the number of non-isomorphism topologies generated from 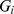 is also increasing. It means that more and more different network topologies appear with the increase of 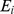, also the corresponding 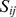. It is necessary to emphasize no matter how many times this network is executed the switching procedure for, the ensemble of all possible non-isomorphism networks share the same in-degree and out-degree sequence with 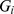. Therefore, when *n* is large enough, 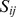 can enumerate all possible 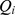. That is to say, at this time, 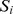 reaches its maximum value 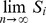. In fact, the metric 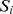 can also be replaced by enumerating non-isomorphism topologies in the set {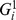, 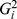
…, 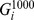}, but it has to face the NP problem: the isomorphism identification of graphs.

Referring to the threshold of detection range of motif identification, the evolutionary process of the Kalundborg case in the interval 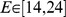 which covers the lower boundary of subgraph template 1 at α = 0.1 is cut out to illustrate the necessity and accuracy of the proposed method.

**Figure 7:**
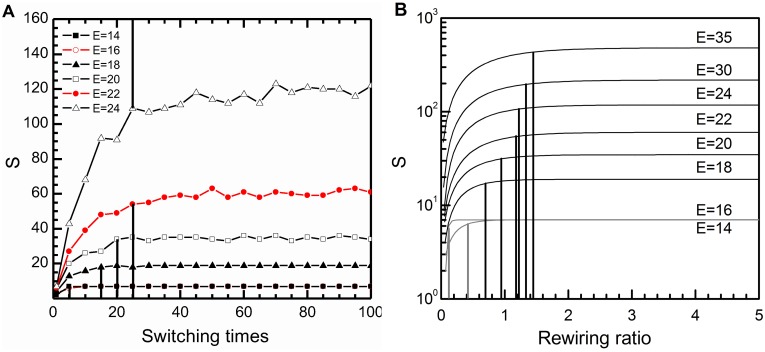
Variation of 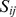 in the evolutionary process of the industrial network at Kalundborg. (**A**) The relationship between the switching times *n* and 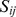 at *E* = 14, 16, 18, 20, 22, 24. (**B**) The relationship between the rewiring ratio *r* (*n*/*E*) and 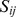 at *E* = 14, 16, 18, 20, 22, 24, 30, 35. The exponential function 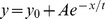 is used to approximate the curve of randomized process of the network 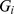 at each time point. And the curves at *E* = 14, 16 are marked in gray.

With the increase of *n*, 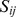
at the time points (*E* = 14, 16, 18, 20, 22, 24) are compared and shown in Figure 7(A), (B). It is concluded that the curvature of each curve decreases gradually and converges to a constant. This constant represents the maximum value of 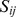. The most distinct difference among these curves is how many switching times it costs to reach to the extreme value 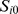. It seems a little hard to distinguish when the network 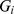 is adequately randomized. In our research the exponential function 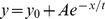 is adopted to approximate these curves. Stipulate that if 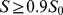, the network 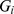 is thought to be adequately randomized. Then the critical switching times 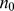 and rewiring ratio 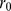 (dividing *n* by *E*) of each curve can be calculated and marked in Figure 7(A), (B). When *E* = 14, the network is adequately randomized just by executing the switching times twice, but later, 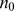 increases to over twenty. From the rewiring ratio point of view, when *E* = 14, 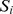 approaches to its maximum value rapidly only by rewiring 13% of the edges, and for *E* = 16, a little better, 42%. For the time interval 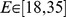, 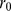 exceeds 100% rapidly. These changes indicate that the result of motif identification before *E* = 18 is quite easily influenced by tiny disturbances and it is not trustworthy. However, with the increase of 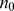 and 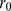, the reliability of results improve significantly. Therefore, it is concluded that the threshold of the detection range proposed in the method of motif identification is reasonable and necessary, especially for those small scale networks, such as food webs, social networks and industrial networks.

## Discussion

Network motifs emerge from the evolutionary process of systems, and meanwhile, grow up to the overrepresented subgraphs. Therefore, it is quite an interesting and important question how shall we distinguish subgraphs which have the potential to be network motifs from common ones, especially in the initial stage of evolving networks. In other words, it is necessary to define the threshold of network size for the detection of motifs. The approximate solutions to the expected value of the appearances of subgraphs in an ensemble of randomized networks, characterized by arbitrary degree sequence, have been given. However, the irregular degree sequences of each real network deviating from standard degree distribution types significantly increase the calculation account. This then leads to the difficulty to define the universal threshold for all networks along the lines proposed in our research. It seems likely that the ratio 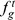 may exceed the interval [0.9, 1.1] for several times at different time points of real evolving networks. Based on the exact equations for the concentrations of all subgraphs in the E-R random network model, this question is simplified into evaluating the relations of two metrics, *N* and *E* of real networks for each subgraph. The corresponding answer should be effective and acceptable to most networks except for those extreme heterogeneous structures, such as star nets.

In the detection of motifs in small networks on time scale, the result at single time point may cause the false appearance of network motifs without considering continuity. The emergence of motifs is generally thought to be caused by optimal design, duplication behavior, or structural preference of the evolutionary process of systems. These important principles are usually unknown, especially in the beginning of evolution, or just assumed by researchers, and needed to be verified by more experiments and data. To decrease false alarm rate of motif detection, it is necessary to expand the definition of traditional measurements of statistical significance to that reflecting the average level in a continuous period of time. For subgraphs whose appearance is just over the frequency threshold of motif, it is particularly important, either in small scale networks or huge networks [41].

The conclusion of lower and upper boundaries of motif detection in growing networks is deduced by E-R random network model. There are also some other kinds of network models to describe the degree distribution of real network, such as scale-free model, small-world model, and regular network model. Each of the structural characteristics of these three network models is more complex than that of E-R random model. For the scale-free model of directed networks, the power exponent of the in-degree and out-degree data may be different, and their values can vary in a relatively wide range in the set of positive real number. In addition, the starting point of fat tail in some common scale-free models could also be different. Because there are more hub vertices in scale-free networks, they could seriously affect the frequency of subgraphs around them, When a new connection is added with obvious preference attachment, it is probably that the change ratio of the frequency of some subgraphs will be very large. Thus, the corresponding lower boundary is supposed to be higher than that of E-R random model, contrary to the conclusion of the upper one. Of course, more accurate results of scale-free model should be proved by strict theoretical analysis and computer simulation experiments.

## Conclusion

In many disciplines, motifs are expected to bridge the communication gap between elementary components and macro properties of networks, such as degree distribution. Thus, by investigating emergence of network motifs, it should be an important perspective to explore and uncover organization rules and evolution mechanism of different systems. The initial growth period of networks could be changeable and many statistical characteristics tend to be stabilized gradually. Just in this special period, the transition of common subgraphs to motifs could be captured and mechanisms behind them become clear. Our research contributes to the traditional methodology of motif identification, which can help us to reject those pseudo motifs and find more robust results. Although only the directed networks are considered in our research, the method for undirected networks can be easily deduced with the same idea.
